# Pure Sensory Thalamic Stroke Presenting As Hemiballismus: A Case Report

**DOI:** 10.7759/cureus.68708

**Published:** 2024-09-05

**Authors:** Bridgette King, Hamza Jamil, Anushka Dekhne, Danial Bajwa, Justin Nolte, Paul Ferguson, Syed Hashim Ali Inam

**Affiliations:** 1 Neurology, Marshall University Joan C. Edwards School of Medicine, Huntington, USA; 2 Internal Medicine, Army Medical College, Rawalpindi, PAK; 3 Internal Medicine, American University of Antigua, St. John's, ATG; 4 Internal Medicine, Combined Military Hospital Rawalpindi, Rawalpindi, PAK

**Keywords:** acute hemichorea, and transient ischemic attack (tia), community stroke, hemichorea-hemiballismus, ischemic cva, thalamus stroke

## Abstract

Strokes are a major cause of morbidity and mortality across the globe. An ischemic stroke of thalamic origin should be considered if a patient presents with a set of non-localizing symptoms such as speech issues, sensory abnormalities, chorea-like movements, ataxia and confusion that cannot be explained by a single lesion. A 78-year-old female with a past medical history of hypertension and smoking developed right-hand numbness and ataxia that progressively worsened to numbness of the entire right side of the body and right-arm hemiballismus. Magnetic resonance imaging (MRI) confirmed an acute left thalamic ischemic stroke as the cause of her symptoms. Our case report highlights the rare clinical presentation of thalamic strokes that can aid in the diagnosis and localization of such pathologies. Further research regarding the best therapy for these post-stroke movement pathologies is needed.

## Introduction

The function of the thalamus is to work as the relay center of the brain, involved in transferring complex brain signals to almost every region of the brain [1]. The thalamus is involved in functions such as strengthening memory, regulating emotions, and controlling sleep-wake patterns. It also supports executive functions, coordinating cortical alertness and processing and transmitting sensory information related to taste, somatosensorial, vision, and audition [1]. In addition, the thalamus assumes a pivotal role in the coordination of sensory-motor integration, thus making a substantial contribution to the smooth performance of motor actions [1]. The significant role of the thalamus in the complex network of brain processes underlying human consciousness and behavior is further highlighted by its extensive involvement [1]. It is frequently noted that thalamic strokes occur either independently or in combination with infarcts that damage other structures [1]. The functional complexity of the thalamic nuclei and the commonly noted normal variations in the arteries supplying the thalamus contribute to the considerable disparities in the genesis of thalamic infarcts [1].

When a patient presents with an unusual set of anomalies that cannot be explained by a single lesion, thalamic pathology should be considered, particularly if awareness is impaired [2]. Thalamic illness might resemble a number of different neurological conditions [2]. The functional complexity of the thalamus and the commonly noted normal variations in the arteries supplying it contribute to the considerable disparity in the genesis of thalamic infarcts and their subsequent clinical presentations [2]. When a patient presents with an unusual set of neurological deficits that are not easily explained by a single lesion, pathology of the thalamus should be considered, particularly if awareness is impaired [2].

Involuntary movements occur in 1-4% of cases after ischemic or hemorrhagic strokes and are caused by damage to the basal ganglia, thalamus, and/or their connections. The most common symptoms among stroke survivors are dystonia in children and hemichorea-hemiballism in adults. Tremor, myoclonus, asterixis, stereotypies, and vascular parkinsonism are a few examples of movement disorders that can occur after a stroke. The onset of these movement disorders can occur in the subacute or chronic periods following the strokes, and the symptoms can sometimes be progressive. Acute strokes can cause some conditions to arise shortly after the event, while others may have a delayed onset and a progressive course. Neural plasticity, age-related changes in brain metabolism, and functional diaschisis are some of the pathophysiological mechanisms suggested to explain this variability in presentation and clinical course [3].

## Case presentation

A 78-year-old female with a past medical history of hypertension, sciatica, and a 25-year history of smoking presented to the hospital with initial right-hand numbness and incoordination that progressively worsened to include numbness in the entire right face, arm, and leg, leading to her presentation to our emergency reception (ER). Symptoms started 26 hours prior to the arrival in the ER, therefore she was not in the window for thrombolysis or thrombectomy.

Initial examination revealed sensory deficits in the right arm and leg, with the patient experiencing difficulty holding objects as she could not maintain the grip strength reflecting a lack of control with the motor planning. Notably, the patient complained of her right arm occasionally striking her face or causing her to drop items which was consistent with right-arm hemiballismus. The patient stated her right arm would thrash up and down without any provocation, occurring multiple times throughout the day, leading to spilling coffee and dropping her food. On one occasion, the abnormal movements led to the patient striking her arm on the railing of the bed, leading to bruising throughout her right arm. When asked, the patient stated she felt as if her arm had a mind of its own. The reflexes were normal in the bilateral lower and upper extremities. Plantar reflex was down going bilaterally, and Hoffman's signs were negative. Sensations on the left side of the body were normal. Finger-to-nose testing on the right was uncoordinated, but it was normal in the left arm. Heel-to-shin testing was slowed in the right leg but it was normal in the left leg. No visual deficits and cranial nerve deficits were observed. Her presenting systolic blood pressure was 157/70, and the patient's home medications of metoprolol and valsartan were continued the following day after permissive hypertension was practiced. Aspirin and atorvastatin were started. Her stroke workup included electrocardiography (EKG), vascular imaging, and an echocardiogram. Vascular assessment revealed minimal stenosis in the right and left common carotid arteries and minimal stenosis in the right and left internal carotid arteries. The vertebral arteries were widely patent. There were no acute hemorrhages, aneurysms, or acute large vessel infarctions observed. EKG and echocardiogram were unremarkable.

**Figure 1:**
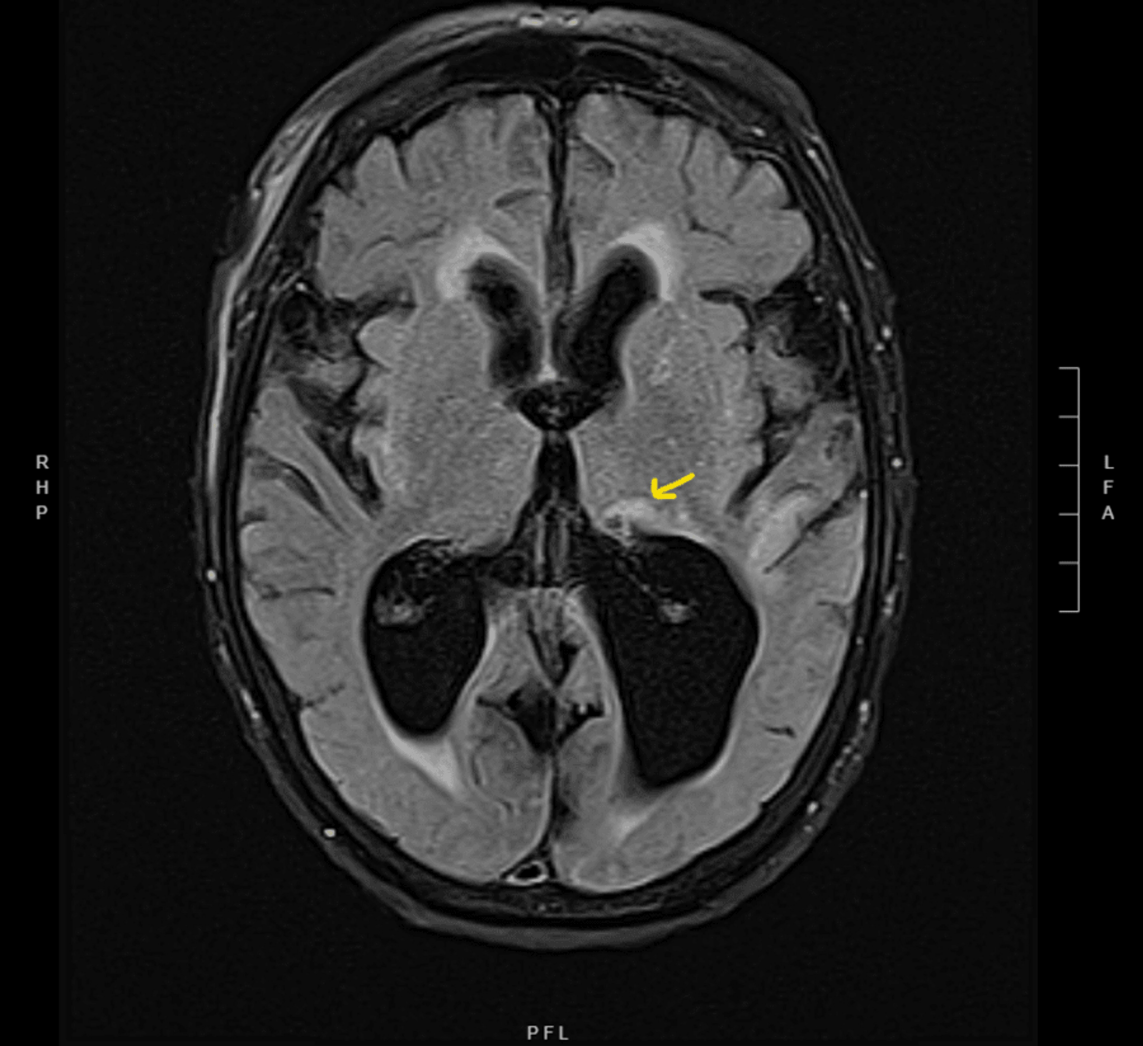
The T2-FLAIR of MRI brain showing hyperintensities in the left thalamic region. There is parenchymal volume loss and nonspecific scattered white matter alteration likely attributed to chronic microangiopathy. FLAIR: fluid-attenuated inversion recovery

She eventually had an MRI that confirmed an acute left thalamic ischemic stroke as the cause of her symptoms. The T2-weighted-fluid-attenuated inversion recovery (FLAIR) MRI image is shown in Figure 1.

She was evaluated by physical and occupational therapy. She was recommended for acute rehab at discharge. Inpatient rehabilitation was planned. Social history included a one-pack per day history of smoking cigarettes for a total of 25 years which is the major contributor to stroke pathology in this case along with hypertension. The patient's history further revealed no other cardiovascular risk factors, such as coronary artery disease, diabetes mellitus, or previous strokes. She denied experiencing weakness, visual disturbances, or balance issues. She was not on blood thinners, aspirin, or a statin, and her blood pressure was reported to be within normal limits at home. The onset of symptoms occurred while the patient was studying, initially manifesting as right-sided hand numbness in the evening. Subsequently, the numbness progressed to involve the entire right side of her body, and this was followed by impaired hand coordination, a novel symptom for the patient. This was the time when the patient decided to go to the ER.

## Discussion

This case illustrates key clinical aspects of a 78-year-old female patient with a left thalamic stroke, presenting with sensory deficits affecting the right arm, leg, and face, with choreiform/hemi ballistic movements in the right arm. Left hemisphere strokes are prevalent in hospital settings, influenced by recognition biases due to the ability of patients to recognize symptoms like aphasia and apraxia over neglect and visuospatial deficits typically seen in right-hemispheric strokes. Thalamic strokes vary in ranges of symptoms based on stroke location, volume, and lateralization, creating complex manifestations linked to affected thalamic nuclei and vascular territories [4]. Only 0.4-0.54% of thalamic strokes present as hemiballismus [4]. Hemiballismus is a rare hyperkinetic disorder that is defined as involuntary, high amplitude, slow frequency thrashing movement of unilateral arm and leg. This is usually seen in the contralateral limb, when an injury to the subthalamic nucleus and basal ganglia is observed [4]. Considering the rarity of this presentation, it is important to emphasize that thalamic strokes can present as hemiballismus of the contralateral side [4]. Our case presents a case with a similar presentation as this would add to the pre-existing literature and aid clinicians in making the right diagnosis [4].

Recent studies indicate significant longitudinal changes in ipsilesional thalamic volume during acute and subacute stroke phases, impacting long-term recovery and quality of life [5]. Understanding these changes in chronic stroke stages requires consideration of factors including lesion size, aging, gender, and intracortical volume.

The incidence and prevalence of stroke globally affects millions annually with significant mortality and long-term disability implications, particularly in low- and middle-income regions [6]. Thalamic strokes account for a notable proportion of posterior circulation infarcts [7]. The thalamus plays a critical role in sensory and motor functions, emotional processing, and cognitive abilities, making thalamic stroke evaluations crucial for accurate diagnosis and treatment planning [8].

Psychosocial deficits are prevalent among stroke survivors, highlighting the importance of early detection and management to enhance post-stroke recovery, an area where nursing interventions can significantly impact patient outcomes [9]. The diverse functional classes of thalamic nuclei underscore how vascular lesions can lead to varied sensory and behavioral impairments [10]. Studies linking thalamocortical projections to thalamic volume changes highlight age-related cortical shrinkage as a contributing factor, necessitating tailored rehabilitation strategies informed by clinical parameters such as gender, lesion size, and age [11].

Around 90% of strokes can be prevented by controlling the modifiable risk factors. Among these risk factors, 75% of them are behavior-related including poor nutrition, tobacco use, and sedentary lifestyle [12]. Hypertension is the most common stroke risk factor [12]. Tobacco use disorder can significantly increase the risk of stroke [13]. There is a worldwide population-attributable risk of stroke of 12.4% associated with current smoking [13]. Our patient had a history of hypertension and tobacco use disorder that predisposed her to this stroke. There is a need to raise awareness about these risk factors and their relation to stroke pathology [13]. The disparities in stroke incidence and outcomes among racial groups, influenced by factors like smoking, obesity, diabetes, and hypertension, emphasize the need for targeted preventive measures [12,13].

## Conclusions

The presentation of left pure sensory thalamic stroke with right arm hemiballismus highlights the rare clinical complexity of ischemic strokes especially thalamic in origin, as they can present with chorea-like movements and impaired awareness. Though rare, this presentation can guide clinicians with the localization of the stroke pathology. The patient presented has a history of hypertension and tobacco use disorder that significantly increased the risk of having a stroke. Our patient's case highlights the importance of in-depth neuroimaging and comprehensive risk factor evaluations in understanding stroke pathophysiology to guide treatment strategies for stroke prevention. Further research and clinical efforts are necessary to determine the underlying mechanisms of thalamic stroke-related movement pathologies and tailor rehabilitation techniques to individual patient needs, taking into account factors such as gender, lesion characteristics, and age-related changes in thalamic volume. Raising awareness about stroke presentations, timely arrival to the hospital, better blood pressure control, and smoking cessation will aid in better stroke management.
